# The General Practitioners and Dr. Rentoul's Scheme

**Published:** 1889-11-23

**Authors:** 


### The General Practitioners and Dr. Rentoul's Scheme.

The South London branch of the British Medical Association met on Wednesday evening, the 13th inst., to discuss the public medical service scheme suggested by Dr. Rentoul. Invitations were issued to general practitioners, which resulted in a fairly good gathering in the handsome board-room at Bethlehem Hospital.

Mr. Nelson Hardy did not agree with Dr. Rentoul's scheme, but had undertaken to bring the matter before the meeting. The existence of hospital abuses was generally admitted. Sir Andrew Clark and others attempted to defend those charities by calling reformers crotchet-mongers and robbers of hospitals. There was, however, an overwhelming mass of evidence to the contrary. Enquiry at St. Thomas's and other hospitals showed a large percentage of abuse. It is of no use looking for help to the surgeons, physicians, or lecturers of the schools; their only eye is to clinical instruction or to personal advantage. Treatment by a bottle of medicine once a week was simple quackery, and yet patients might be seen dragging up to consumption hospitals to be so treated month after month. In view of all these points, and of the other evidence at command, the agitators may rest content, although they be called crotchet-mongers and other opprobrious names. The speaker failed to see what benefit was to be gained from organising the hospitals, and asked pertinently if those institutions were likely to do right together where they had sinned individually. As to securing their co-operation in the new scheme, that was a problem for Dr. Rentoul himself to solve. One great difficulty was the supply of material for medical students. In discussing proposed changes, he mentioned incidentally that no instrument maker in London would think of sending his unqualified assistant to fit on a poroplastic jacket under half a guinea. His idea was rather to bring existing dispensaries up to the level of Poor Law infirmaries. As to the proposed alliance with chemists it seemed a degree worse than a sixpenny dispensary, which was held to disqualify from membership of that association. Dr. Rentoul seemed to have forgotten the existence of the Poor Law service, by which an enormous amount of relief is effected.

Mr. Campbell Boyd, a member of the branch, then moved the following resolution on behalf of Mr. Hentsch, one of the visitors, who were allowed to speak but not to vote: "That this meeting considers Dr. Rentoul's scheme would be impracticable, and that, even if it were practicable, it would be disadvantageous to the great bulk of the general practitioners." Dr. Oldfield seconded.

Mr. Hentsch regarded Dr. Rentoul's proposal as introducing another competing body into our midst, which could only result in still further lessening the remuneration of the profession. It is proposed to map out the country into districts, each of which will be handed over to a knot of general practitioners, who will then pose before the public as a superior class. The scheme is admittedly imperfect, as it does not provide for those who are too ill to leave home, nor for midwifery cases. Dr. Rentoul's suggestion that patients ill at home would send for their service doctor, who could then charge his own fee, was simply a direct temptation to the unsuccessful general practitioner to join the scheme for the purpose of taking patients from more successful men. In short, public service was nothing more than the provident dispensary under another form. Dr. Rentoul aims at (1) reform of out-patient departments, (2) prevention of counter prescribing by chemists, (3) removal of sixpenny doctors, (4) abolition of club practice. Mr. Hentsch asserted that the hospitals are over-crowded by people who are perfectly able to pay ordinary medical fees, not by those who are unable. It was absurd to suppose that the mere formation of this service will stop the illegal prescribing of chemists. The speaker vigorously defended the sixpenny doctors: said that the abolition of clubs simply meant their being swallowed up in a medical provident system, and wound up with an eloquent appeal for firm professional union.

Dr. Brindley James agreed with the last speaker as to the needs of co-operation and of union. Scrutiny of patients was a necessity. He approved of Dr. Rentoul's scheme, with certain modifications, based on the working of the South London Medical Aid Society, which had been founded for the poorer class who could not pay small fees, and to which all the old practitioners of Bermondsey belonged. The entire abolition of the out-patient departments would provide plenty of patients for the general practioner. As to lack of material for students, they could easily be taught in the Poor Law dispensaries and infirmaries, where they would have the chance of seeing diseases, the zymotic, for instance, which they did not meet with under present conditions. Students could also attend patients at their own homes. That something was wanted the speaker did not doubt, but in his opinion Dr. Rentoul's scheme was calculated, however imperfectly, to forward that object, and he should therefore oppose the motion.

Dr. Corbyn questioned if the whole scheme of benevolence were not a mistake. He had received a letter from Dr. Rentoul, supposed to be explanatory, but which read like an Athanasian Creed. That gentleman's scheme was no more wanted than the out-patient departments of hospitals, and in his opinion the latter were not wanted one little bit. Special departments, however, dealing with subjects that general practitioners had not time to study, were no doubt a great boon on all hands. He asserted that out-patient practice existed simply and solely, absolutely and entirely, for the sake of a certain limited number of men. Out of the 20,000 or so names on the register, about 18,000 were those of general practitioners. It was a disgrace to the profession that the agitation for a Royal Commission should have been started by the Charity Organisation Society. Now, it remained to get evidence together to lay before that commission. As to the out-patient hospital staffs, everyone would no doubt be sorry to see those gentlemen deprived of their little sinecures. Still, it was a question between 18,000 on the one side and 2,000 on the other. Let there be a fair field and no favour. So far as he (the speaker) could make out, Dr. Rentoul wanted employers to raise the wages of workmen, that the latter might be able to pay more to the doctors. Such an interference between capital and labour would be enough to wreck a ministry.

Dr. Hugh Woods rose with an amendment. He pointed out that the scheme, although wanting in details, had already been considerably modified. A representative body of medical men was wanted to settle such points as prices. Then the whole body of general practitioners should stand shoulder to shoulder. It was not Dr. Rentoul's intention to form a "corner," for any respectable man had the chance of election. There was no particular reason why patients should not be seen at their own homes; indeed, the need of any system at all was not very apparent. Treatment in sixpenny dispensaries was usually bad. Clubs, dispensaries, and other charities were all competing for the poor. Why should not medical men form a firm union and let the poor have their treatment as cheaply as possible? Make those pay who can pay, and who now get off scot-free. A child was brought to him suffering from ringworm. That child was afterwards taken off to a skin hospital, and there charged half-a-crown, whereas his own charge would have been three and sixpence. It was natural for people to go to noted specialists at hospitals, when they could consult them there for less than their own medical men. Dr. Rentoul was ready to take up any other scheme, and invites aid of medical men all over the country.

Dr. Oldfield observed that the damage done by the out-patient departments would be much lessened if each applicant were obliged to get recommended by a practitioner of his district. Medical men would be likely to have the necessary information as to the social position of patients. Everyone in practice should adopt a system of sick assurance with a wage limit.

Dr. Campbell Boyd found, as a general practitioner, that plenty of his patients went to the hospitals because they could get in there for nothing. In his opinion the adoption of a wage limit would lead to a large amount of deception. Medical men form a network over the whole country, and would be well able to recommend those patients who were suitable. Dr. Rentoul's scheme would magnify the few at the expense of the many.

The President said he could quite understand there was ground for a free expression of feeling. Abuses are said to exist in both out and in departments. From such of us as are averse to enquiry, an explanation may be properly asked. The whole question seems to be overrated. Very many cases of patients are not so well known to medical men outside as to those on the staffs. Many who go to hospitals have first spent a certain amount on outside practitioners, and in chronic cases their money is often exhausted. The out-patient room is a valuable means of education for students, whom he would not care to turn loose in Poor Law infirmaries. If out-patient rooms were closed, then there would be no further need of organisation on the part of general practitioners; patients must of necessity go to them.

The resolution condemning Dr. Rentoul's scheme was carried by a large majority—a result on which the general practitioners present congratulated each other warmly.

